# A Novel Role of Serotonin Receptor 2B Agonist as an Anti-Melanogenesis Agent

**DOI:** 10.3390/ijms17040546

**Published:** 2016-04-12

**Authors:** Eun Ju Oh, Jong Il Park, Ji Eun Lee, Cheol Hwan Myung, Su Yeon Kim, Sung Eun Chang, Jae Sung Hwang

**Affiliations:** 1 Department of Genetic Engineering, Graduate School of Biotechnology, Kyung Hee University, Yongin 446-701, Korea; singey@naver.com (E.J.O.); rystrong@khu.ac.kr (J.I.P.); jieun0405@khu.ac.kr (J.E.L.); audjoin2@naver.com (C.H.M.); 2 Department of Dermatology, Asan Medical Center, University of Ulsan College of Medicine, 388-1 Pungnap-dong Songpa-gu, Seoul 138-736, Korea; u2u2star@naver.com

**Keywords:** BW723C86, melanogenesis, PKA, CREB, MITF

## Abstract

BW723C86, a serotonin receptor 2B agonist, has been investigated as a potential therapeutic for various conditions such as anxiety, hyperphagia and hypertension. However, the functional role of BW723C86 against melanogenesis remains unclear. In this study, we investigate the effect of serotonin receptor 2B (5-HTR2B) agonist on melanogenesis and elucidate the mechanism involved. BW723C86 reduced melanin synthesis and intracellular tyrosinase activity in melan-A cells and normal human melanocytes. The expression of melanogenesis-related proteins (tyrosinase, TRP-1 and TRP-2) and microphthalmia-associated transcription factor (MITF) in melan-A cells decreased after BW723C86 treatment. The promoter activity of MITF was also reduced by BW723C86 treatment. The reduced level of MITF was associated with inhibition of protein kinase A (PKA) and cAMP response element-binding protein (CREB) activation by BW723C86 treatment. These results suggest that the serotonin agonist BW723C86 could be a potential therapeutic agent for skin hyperpigmentation disorders.

## 1. Introduction

Although melanin protects the skin from ultraviolet (UV) radiation, an imbalance in melanin synthesis causes pigmentary disorders [1,2,3]. Hypermelanosis, particularly that affecting the face and neck, can cause marked cosmetic disability and mental distress. A number of skin whitening agents have been used but acceptable safety and efficacy has not been achieved. Ideal skin whitening agents should enhance skin integrity and health and not merely bleach the skin, which is often associated with skin irritation and other long-term adverse events [4].

Serotonin (5-hydroxytryptamine, 5-HT) has been one of the most popular candidates for enhancing human health although the scientific evidence is inconclusive. Serotonergic abnormalities and serotonin receptors have been studied in the brains of adult suicide victims [5]. BW723C86, a serotonin receptor 2B agonist, has been reported to have therapeutic effects on conditions such as anxiety, hyperphagia and hypertension [6,7,8]. Serotonin is produced by a multistep metabolic pathway. l-tryptophan is hydroxylated by tryptophan hydroxylase, which is expressed in mouse, hamster and human skin [9,10,11]. Hydroxytryptophan is then decarboxylated to produce serotonin [12,13]. Serotonin regulates many physiological processes such as vasoconstriction, stress responses, and sexual desire [14] through mechanisms that are dependent on or independent of a family of seven receptors (5-HTR1-7). Diverse serotonin receptors are expressed depending on the cell type. Genes encoding 5-HTR1A, 1B, 2A, 2B, 2C, and 7 have been identified in skin cells [15,16]. Among these, the 5-HT2B and 5-HT7 genes are expressed in both human and rodent skin cells [17,18]. Previous studies have shown that serotonin inhibits melanin synthesis in human melanoma cells. Other studies, however, reported that serotonin induces melanogenesis through serotonin receptor 2A in SK-MEL-2 melanoma cells [19,20]. These effects might be exerted by melatonin and its metabolites produced from serotonin in melanoma cells [20,21].

**Figure 1:**
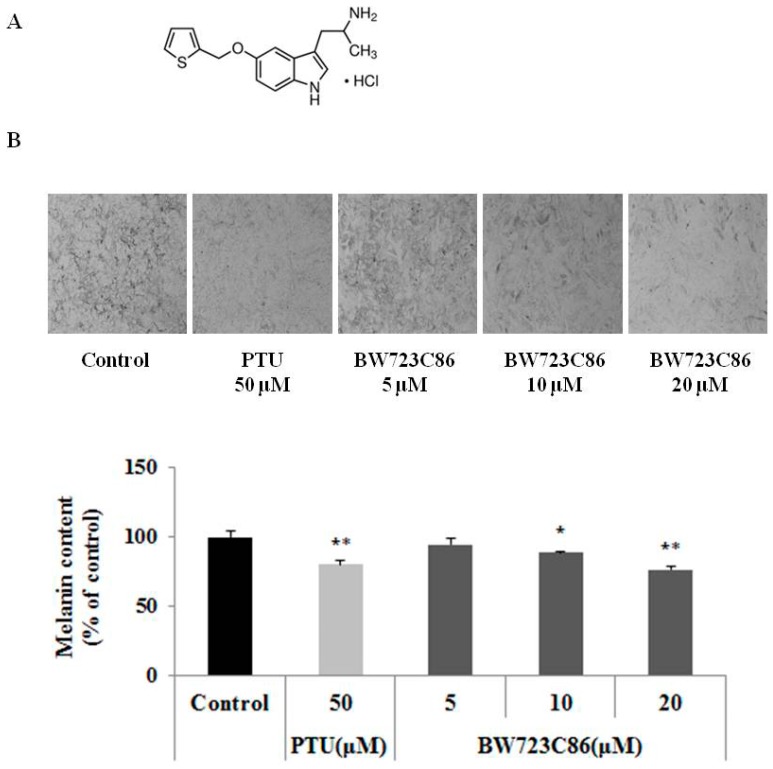

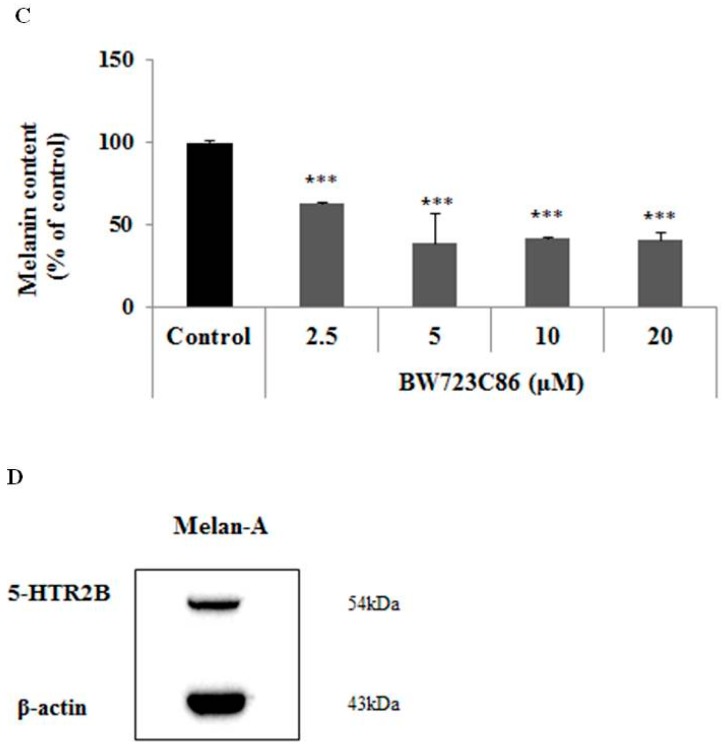
The effect of BW723C86 on melanin synthesis in melan-A cells and human melanocytes and the expression of serotonin receptor 2B in melan-A cells. (**A**) The structure of BW723C86 (α-methyl-5-(2-thienylmethoxy)-1H-indole-3-ethanamine); (**B**) Bright-field image (×100) of melanocytes and results of melanin assay in melan-A cells treated with BW723C86 for 72 h; (**C**) Melanin assay in human melanocytes treated with BW723C86 for 72 h. Phenylthiourea (PTU) was used as a positive control; (**D**) Expression of serotonin receptor 2B (5-HTR2B) was measured by Western blotting. β-actin was used as a protein loading control. Values are means ± SD from three replicates (*n* = 3). Statistical significance was determined by the Student *t*-test (* *p* < 0.05, ** *p* < 0.01, *** *p* < 0.001).

To date, the effect of serotonin and role of the serotonin receptors in melanogenesis are unclear. Thus, we hypothesized that serotonin is important for maintenance of skin integrity, and particularly in the regulation of melanogenesis. Although serotonin itself did not alter melanogenesis in melan-A cells, we found that the serotonin receptor 2B agonist BW723C86 (α-methyl-5-(2-thienylmethoxy)-1*H*-indole-3-ethanamine) (Figure 1A) profoundly reduced melanin synthesis. In the current study, we further investigated the effects of BW723C86 on melanogenesis and sought to determine the underlying mechanism of action and the role of serotonin receptor 2B.

## 2. Results and Discussion

### 2.1. Effect of BW723C86 on Melanin Content in Melan-A Cells and Human Melanocytes

When melan-A cells and normal human melanocytes were treated with BW723C86 for 72 h, the melanin content was reduced in a dose-dependent manner in all cells (Figure 1B–D). We also confirmed the protein expression of serotonin receptor 2B (5-HTR2B) in melan-A cells (Figure 1D).

### 2.2. Effect of BW723C86 on Tyrosinase Activity in Melan-A Cells

**Figure 2:**
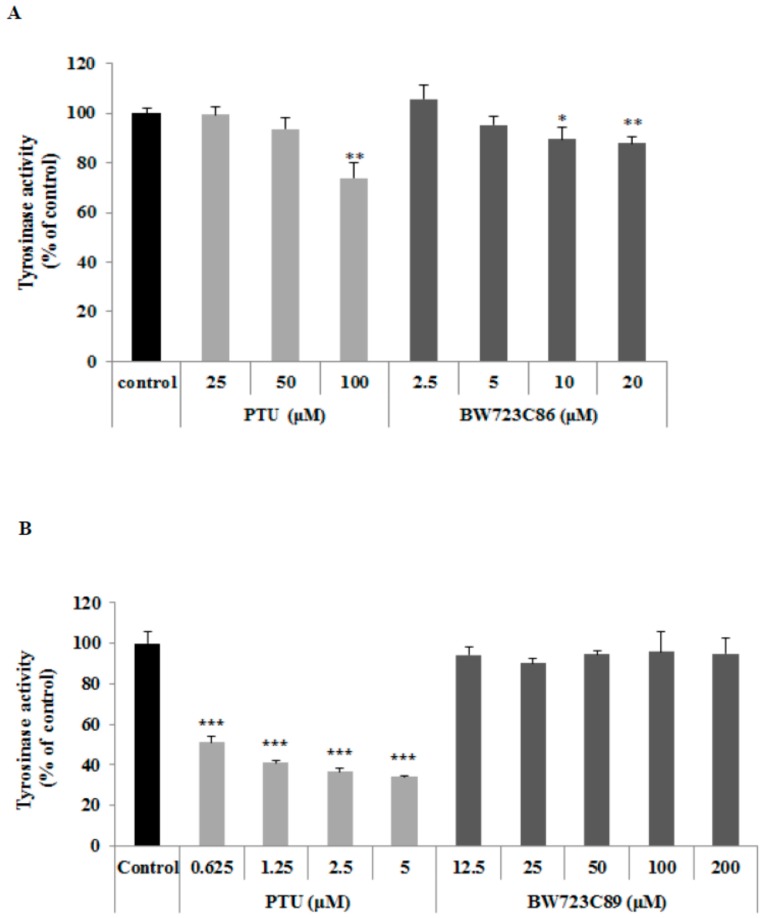
The effect of BW723C86 on tyrosinase activity in melan-A cells determined by measuring the rate of dopachrome formation from l-3,4-dihydroxyphenylalanine (l-DOPA). (**A**) Intracellular tyrosinase activity; (**B**) Tyrosinase activity in cell extract. PTU was used as a positive control. Values are the means ± SD from three replicates (*n* = 3). Statistical significance was determined using the Student *t*-test (* *p* < 0.05, ** *p* < 0.01, *** *p* < 0.001).

To determine the effect of BW723C86 on tyrosinase activity, we measured intracellular tyrosinase activity in melan-A melanocytes. To measure the direct inhibitory effect on tyrosinase, we also measured tyrosinase activity in cell extracts. BW723C86 treatment decreased intracellular tyrosinase activity (Figure 2A), but did not affect tyrosinase activity in the cell extract (Figure 2B).

### 2.3. Effect of BW723C86 on the Expression of Melanogenesis-Related Proteins and Microphthalmia-Associated Transcription Factor (MITF)

**Figure 3:**
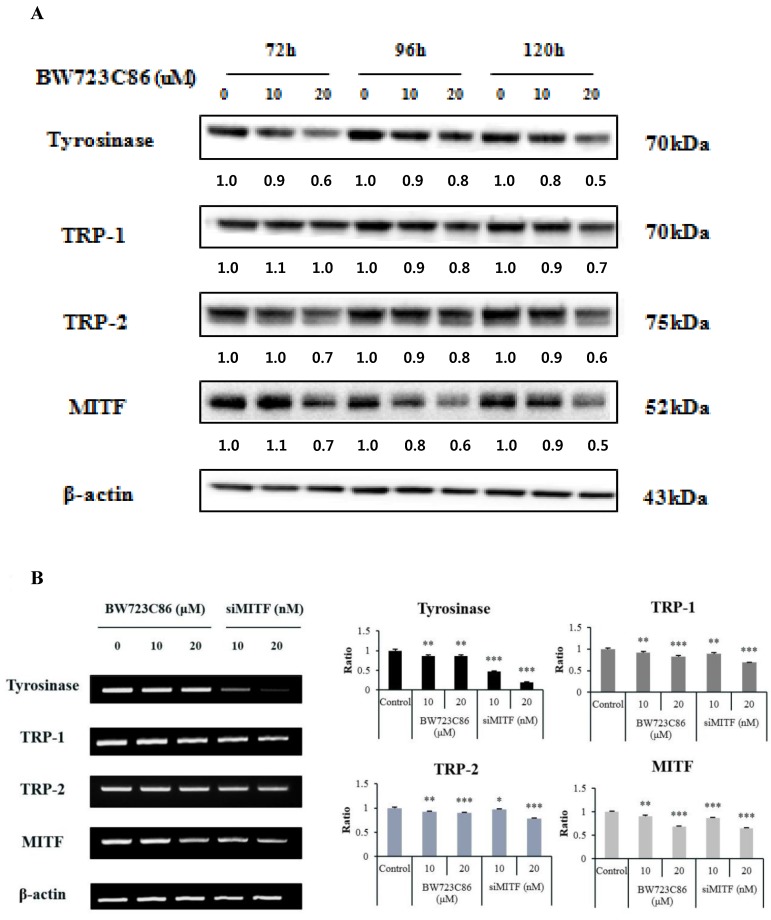
The effect of BW723C86 on the expression of melanogenesis-related proteins and microphthalmia-associated transcription factor (MITF) in melan-A cells. (**A**) Protein levels of melanogenesis-related proteins (tyrosinase, TRP-1, and TRP-2) and MITF were measured by Western blotting after treatment with BW723C86. β-actin was used as a protein loading control; (**B**) mRNA levels of melanogenesis-related proteins and MITF were measured by RT-PCR. MITF siRNA (siMITF) was used as a positive control. The band intensity was normalized to β-actin using the Image J program (National Institute of Health, Bethesda, MD, USA). Values are the means ± SD from three replicates (*n* = 3). Statistical significance was determined using the Student *t*-test (* *p* < 0.05, ** *p* < 0.01, *** *p* < 0.001).

To determine the effect of BW723C86 on the expression of melanogenesis-related proteins (tyrosinase, TRP-1 and TRP-2) and microphthalmia-associated transcription factor (MITF) in melan-A melanocytes, we performed Western blot analysis after BW723C86 treatment for 72, 96, and 120 h. BW723C86 suppressed the protein levels of tyrosinase, TRP-1, TRP-2, and MITF in a dose-dependent manner at each time point (Figure 3A). BW723C86 also reduced the relative mRNA levels of tyrosinase, TRP-1, TRP-2, and MITF at 36 h (Figure 3B).

### 2.4. BW723C86 Downregulated the Protein Kinase A (PKA)/cAMP Response Element-Binding Protein (CREB)/MITF Signaling Pathway in Melan-A Cells

**Figure 4:**
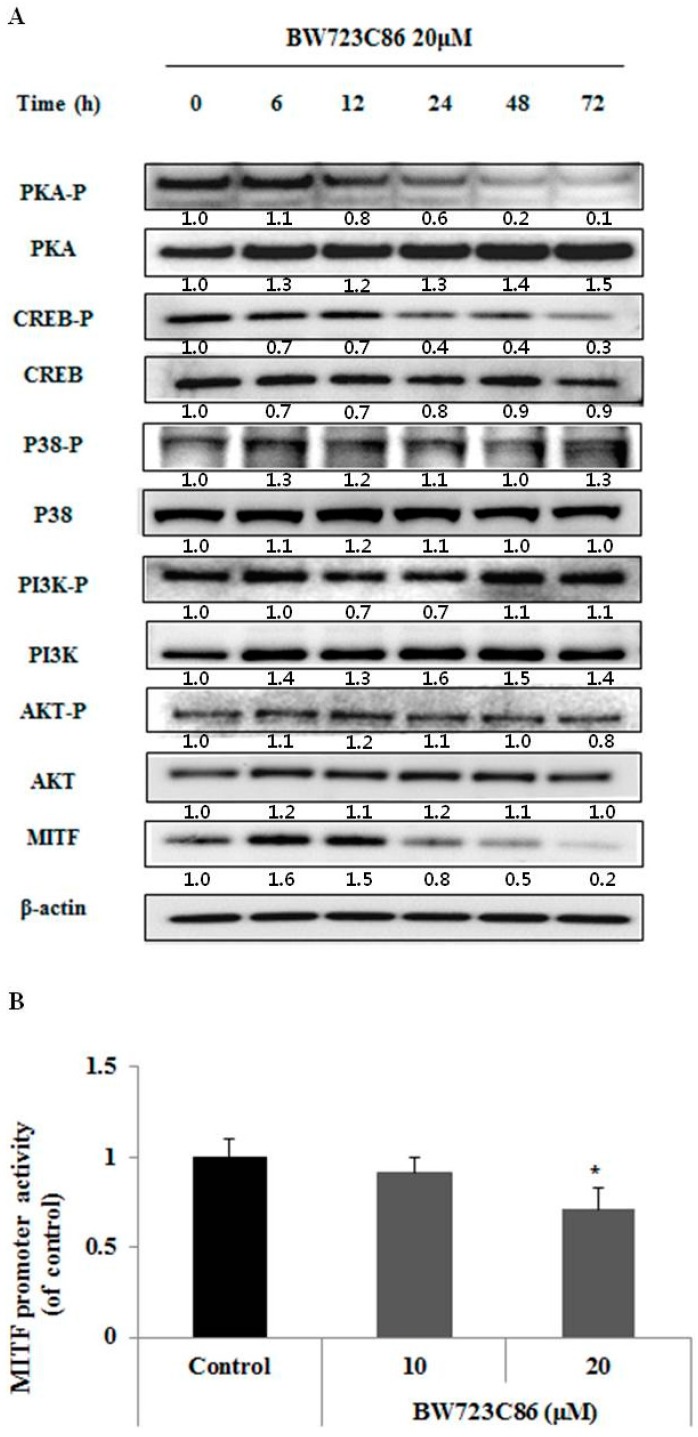
Effect of BW723C86 on the phosphorylation of phosphatidylinositol 3-kinase (PI3K)/AKT, p38 MAPK, protein kinase A (PKA), and cAMP response element-binding protein (CREB), and on MITF promoter activity. (**A**) Changes in the protein expression of MITF and expression and phosphorylation of PI3K, AKT, p38 MAPK, PKA, and CREB were measured by Western blotting. β-actin was used as the protein loading control; (**B**) Melan-A cells containing pMITF-GLuc were cultured for 24 h with BW723C86. *Gaussia* luciferase activity was determined from culture supernatants using the *Gaussia* Luciferase Assay Kit. Values are means ± SD from three replicates (*n* = 3). Statistical significance was determined using the Student *t*-test (* *p* < 0.05).

BW723C86 treatment decreased the phosphorylation of protein kinase A (PKA) and cAMP response element-binding protein (CREB) in a time-dependent manner; however, the phosphorylation of phosphatidylinositol 3-kinase (PI3K)/AKT, and P38 was not changed (Figure 4A). To confirm the effect of BW723C86 on MITF, we showed that BW723C86 significantly decreased the activity of the MITF promoter at a concentration of 20 μM (Figure 4B).

## 3. Experimental Section

### 3.1. Cell Culture

Melan-A cells provided by Dorothy Bennett (St. George’s Hospital, London, UK) are an immortalized normal murine melanocyte cell line derived from C57BL/6 mice. Cells were cultured in RPMI1640 (Welgene, Daegu, Korea) supplemented with 10% Fetal Bovine Serum (FBS) (Welgene), 1% penicillin/streptomycin, and 200 nM phorbol 12-myristate 13-acetate (PMA) (Sigma, St. Louis, MO, USA) at 37 °C, 10% CO_2_. Normal human melanocytes (passage 8) were cultured in medium 254 with melanocyte growth factor supplement (Invitrogen, Carlsbad, CA, USA).

### 3.2. Melanin Assay

After treatment with BW723C86 for 72 h, cells were dissolved in 1 N NaOH at 55 °C for 30 min and absorbance of the cell lysates was analyzed at 490 nm using a spectrophotometer (TECAN, Stockholm, Switzerland). The data were normalized to the protein contents of the cell lysates determined using the BCA Protein Assay kit (Pierce Biotechnology Inc., Rockford, IL, USA). Phenylthiourea (PTU), a known tyrosinase inhibitor, was used as a positive control.

### 3.3. Tyrosinase Activity Assay

To test whether BW723C86 inhibits tyrosinase activity directly, we tested its effect in cell lysates. Cells were seeded on 6-well plates (1.5 × 10^5^ cells/well) and cultured for four days. Cells were removed from the plates and lysed in Radio Immuno Precipitation Assay (RIPA) buffer (Noble Bio, Suwon, Korea). The resultant cell extracts were added to 96-well plates in the presence of various concentrations of BW723C86. To begin the enzymatic assay, l-3,4-dihydroxyphenylalanine (l-DOPA) (2 mg/mL) was added to the lysates. After incubation for 1 h, dopachrome formation was assayed by measuring the absorbance at 475 nm using a spectrophotometer. All data were normalized to the protein content of the cell lysates determined using a BCA Protein Assay kit.

To test whether BW723C86 inhibits tyrosinase activity in culture, intracellular tyrosinase activity was determined by measuring the rate of dopachrome formation from l-DOPA. Cells were seeded on 6-well plates (1.5 × 10^5^ cells/well) and cultured for 72 h with various concentrations of BW723C86. Cells were removed from the plates and lysed in RIPA buffer. Cell extracts were added to a 96-well plate and l-DOPA (2 mg/mL) was added. The enzymatic assay was performed as described above.

### 3.4. Western Blot Analysis

The protein content of the supernatant was quantified using a BCA Protein Assay kit with bovine serum albumin as the standard. Equal amounts of protein were separated by NuPAGE^®^ 10% Bis-Tris gel (Invitrogen) and transferred onto polyvinylidene fluoride (PVDF) transfer membranes. The membranes were probed with antibodies against MITF (Thermo Scientific, Rockford, IL, USA), β-actin (Sigma), 5-HTR2B, p-PI3K, PI3K (Abcam, Cambridge, UK), p-PKA, PKA (Santa Cruz Biotechnology, Santa Cruz, CA, USA), p-CREB, CREB, p-AKT, AKT, p-P38, P38 (Cell Signaling Technology, Beverly, MA, USA), horseradish peroxidase-conjugated anti-rabbit (Bethyl, Montgomery, TX, USA) and anti-mouse (Bio-Rad, Hercules, CA, USA) IgG. Antibodies against tyrosinase (aPEP7), TRP-1 (aPEP1), and TRP-2 (aPEP8) were a gift from Vincent J. Hearing (National Institute of Health, Bethesda, MD, USA). Binding antibodies were detected using a WEST-ZOL^®^ Plus Western Blot Detection System (INtRON Biotechnology, Sungnam, Korea).

### 3.5. RT (Reverse Transcription)-PCR

Total cellular RNA was extracted from melan-A cells using Trizol Reagent (Takara, Otsu, Japan). One microgram of total RNA was mixed with 100 pmol oligo d(T) (ELPIS, Daejeon, Korea) and denatured for 5 min at 65 °C. The annealed samples were incubated with reverse transcriptase and 2 mM dNTPs (Fermentas, Hanover, MD, USA) for 1 h at 42 °C. Reverse transcription was terminated by heating for 10 min at 70 °C. cDNA was mixed with HiPi PCR Mix (ELPIS) and each sense and antisense primer (tyrosinase: sense 5′-GCAAAAGAATGCTGCCCACC-3′, anti 5’-ATGTCCCTCCATATTTCAGAGCC-3′, TRP-1: sense 5′-TATTGAGGCTCTGAGACGTGGGG-3′, antisense 5′-CTCCTTGTGGCAATGACAAATTG-3′; TRP-2: sense 5′-TTCAGCACGCCATCCAAGGTCATG-3′, antisense 5′-GAGAAGCTCCCCTCATTAAACCTG-3′, MITF: sense 5′-CGGGTAACGTATTTGCCATTTG-3′, antisense 5′-GCCTGAAACCTTGCTAGGCTGG-3′, β-actin: sense 5′-GTGGGGCTGCCCCAGGCACCA-3′, antisense 5′-CTCCTTAATGTCACGCACGATTTC-3′).

### 3.6. Transient Transfection with siRNA

Melan-A cells were transfected with siRNA using Lipofectamine (Invitrogen) according to the manufacturer’s instructions (MITF siRNA: sense 5′-GACUUUCUCGCUUUCUAAAUU-3′, antisense: 5′-UUUAGAAAGCGAGAAAGUCUU-3′).

### 3.7. MITF Promoter Activity Assay

MITF promoter activity was examined using pMITF-Gluc, which was kindly provided by the AMOREPACIFIC R&D Institute (Yongin, Korea), as described previously [22]. After treatment with BW723C86 for 48 h, cells were lysed in RIPA buffer. *Gaussia* luciferase activity in culture supernatants was determined using a *Gaussia* Luciferase Assay Kit (New England BioLabs, Ipswich, MA, USA). Luciferase activity was normalized to pGLuc-basic activity and the results were expressed as fold stimulation of luciferase activity relative to the unstimulated control.

### 3.8. Statistical Analysis

All experiments were performed three times. The results are expressed as the mean ± standard deviation (SD). Statistical analysis was performed by Student *t*-test.

## 4. Conclusions

Previous studies have indicated that serotonin has vascular, proinflammatory, and pruritogenic effects in the skin. The cutaneous serotoninergic system preserves the biological integrity of skin and maintains its homeostasis to avoid internal or external stress. Although serotonin has a long-suspected association with skin pigmentation, there have been few studies of the relationship between specific serotonin agonists and melanogenesis.

In this study, we demonstrated that BW723C86, a serotonin receptor 2B (5-HTR2B) agonist, is able to reduce skin pigmentation. BW723C86 treatment reduced melanin content in melan-A cells and in normal human melanocytes (NHM) without affecting cellular viability. BW723C86 reduced intracellular tyrosinase activity but did not affect tyrosinase activity in cell extracts, indicating that BW723C86 does not have a direct effect on tyrosinase activity.

MITF is a master transcription factor of three major pigmentation enzymes (tyrosinase, TPR-1, and TRP-2) [23]. MITF binds the M-box motif of tyrosinase, TRP-1, and TRP-2 to activate protein expression [24]. We found that BW723C86 decreased RNA and protein expression levels of MITF. These findings indicate that the protein levels of tyrosinase, TPR-1, TRP-2, and MITF were decreased as a result of downregulation of MITF mRNA levels in response to BW723C86 treatment.

Many studies have identified that protein kinase A (PKA) has four isoforms of the regulatory subunit (RIα, RIβ, RIIα and RIIβ) and three isoforms of the catalytic subunit (Cα, Cβ, Cγ). When cAMP combines with regulatory subunits of PKA, the catalytic subunits are released. Free PKA catalytic subunits translocate to the nucleus and phosphorylate cAMP response element-binding protein (CREB) at serine 133. The phosphorylated active form of CREB binds the cAMP response element (CRE) motif located in the MITF promoter and thus regulates MITF gene expression [25,26,27]. P38 MAP kinase signaling also contributes to CREB activation by phosphorylation of serine 133 [28]. Previous studies have shown that inhibition of AKT, which is downstream of phosphatidylinositol 3-kinase (PI3K), results in activation of glycogen synthase kinase 3β (GSK3β), which in turn enhances the binding of MITF to the tyrosinase promoter [29,30]. Therefore, we examined how MITF transcription is regulated by BW723C86. The phosphorylation of PI3K, AKT, and p38 was unchanged by BW723C86 treatment; however, BW723C86 reduced the expression of MITF and the phosphorylation of PKA and CREB. More importantly, BW723C86 decreased MITF promoter activity. These results indicate that BW723C86 directly inhibits the PKA/CREB/MITF pathway leading to decreased expression of melanogenic proteins.

The effect of serotonin on melanogenesis is unclear. Lee *et al.*, showed that serotonin induces melanin production in melanocytes and melanoma cells [19]; however, Slominski *et al.*, found that serotonin inhibits melanogenesis in human SK-MEL-188 melanoma cells [6]. Our study could help us understand of the role of serotonin in melanogenesis.

In summary, results of our study indicated that BW723C86 inhibits melanin synthesis by suppressing the expression of melanogenesis-related proteins (tyrosinase, TRP-1, and TRP-2) at the transcriptional level. The decreased expression of these proteins is a result of reduced MITF expression. Furthermore, the reduced level of MITF was associated with inhibition of the PKA/CREB/MITF pathway and direct inhibition of MITF transcription. We therefore identified a novel role of a specific serotonin agonist as an anti-melanogenesis agent. Although serotonin itself may show contradictory results depending on cell types this specific agonist might be used for the treatment of hyperpigmentation.

## References

[1] Yamaguchi Y., Brenner M., Hearing V.J. (2007). The regulation of skin pigmentation. J. Biol. Chem..

[2] Yamaguchi Y., Hearing V.J. (2009). Physiological factors that regulate skin pigmentation. Biofactors.

[3] Briganti S., Camera E., Picardo M. (2003). Chemical and instrumental approaches to treat hyperpigmentation. Pigment Cell Res..

[4] Kim J.H., Lee S.M., Myung C.H., Lee K.R., Hyun S.M., Lee J.E., Park Y.S., Jeon S.R., Park J.I., Chang S.E. (2015). Melanogenesis inhibition of β-lapachone, a natural product from *Tabebuia avellanedae*, with effective *in vivo* lightening potency. Arch. Dermatol. Res..

[5] Pandey G.N., Dwivedi Y., Rizavi H.S., Ren X., Pandey S.C., Pesold C., Roberts R.C., Conley R.R., Tamminga C.A. (2002). Higher expression of serotonin 5-HT_2a_ receptors in the postmortem brains of teenage suicide victims. Am. J. Psychiatry.

[6] Slominski A., Tobin D.J., Shibahara S., Wortsman J. (2004). Melanin pigmentation in mammalian skin and its hormonal regulation. Physiol. Rev..

[7] Kennett G.A., Bright F., Trail B., Baxter G.S., Blackburn T.P. (1996). Effects of the 5-HT2B receptor agonist, BW 723C86, on three rat models of anxiety. Br. J. Pharmacol..

[8] Kennett G.A., Ainsworth K., Trail B., Blackburn T.P. (1997). BW 723C86, a 5-HT2B receptor agonist, causes hyperphagia and reduced grooming in rats. Neuropharmacology.

[9] Slominski A., Pisarchik A., Semak I., Sweatman T., Szczesniewski A., Wortsman J. (2002). Serotoninergic system in hamster skin. J. Investig. Dermatol..

[10] Slominski A., Pisarchik A., Semak I., Sweatman T., Wortsman J. (2003). Characterization of the serotoninergic system in the C57BL/6 mouse skin. Eur. J. Biochem..

[11] Slominski A., Pisarchik A., Johansson O., Jing C., Semak I., Slugocki G., Wortsman J. (2003). Tryptophan hydroxylase expression in human skin cells. Biochim. Biophys. Acta.

[12] Slominski A., Wortsman J., Tobin D.J. (2005). The cutaneous serotoninergic/melatoninergic system: Securing a place under the sun. FASEB J..

[13] Slominski A., Pisarchik A., Semak I., Sweatman T., Wortsman J., Szczesniewski A., Slugocki G., McNulty J., Kauser S., Tobin D.J. (2002). Serotoninergic and melatoninergic systems are fully expressed in human skin. FASEB J..

[14] Nordlind K., Azmitia E.C., Slominski A. (2008). The skin as a mirror of the soul: exploring the possible roles of serotonin. Exp. Dermatol..

[15] Johansson O., Liu P.Y., Bondesson L., Nordlind K., Olsson M.J., Löntz W., Verhofstad A., Liang Y., Gangi S. (1998). A serotonin-like immunoreactivity is present in human cutaneous melanocytes. J. Investig. Dermatol..

[16] Slominski A.T., Zmijewski M.A., Skobowiat C., Zbytek B., Slominski R.M., Steketee J.D. (2012). Sensing the environment: Regulation of local and global homeostasis by the skin’s neuroendocrine system. Adv. Anat. Embryol. Cell Biol..

[17] Slominski A., Pisarchik A., Zbytek B., Tobin D.J., Kauser S., Wortsman J. (2003). Functional activity of serotoninergic and melatoninergic systems expressed in the skin. J. Cell. Physiol..

[18] Slominski A., Pisarchik A., Wortsman J. (2004). Expression of genes coding melatonin and serotonin receptors in rodent skin. Biochim. Biophys. Acta.

[19] Lee H.J., Park M.K., Kim S.Y., Park Choo H.Y., Lee A.Y., Lee C.H. (2011). Serotonin induces melanogenesis via serotonin receptor 2A. Br. J. Dermatol..

[20] Slominski A., Semak I., Pisarchik A., Sweatman T., Szczesniewski A., Wortsman J. (2002). Conversion of l-tryptophan to serotonin and melatonin in human melanoma cells. FEBS Lett..

[21] Kim T.K., Kleszczynski K., Janjetovic Z., Sweatman T., Lin Z., Li W., Reiter R.J., Fischer T.W., Slominski A.T. (2013). Metabolism of melatonin and biological activity of intermediates of melatoninergic pathway in human skin cells. FASEB J..

[22] Park W.S., Kwon O.S., Yoon T.J., Chung J.H. (2014). Anti-graying effect of the extract of *Pueraria thunbergiana* via upregulation of cAMP/MITF-M signaling pathway. J. Dermatol. Sci..

[23] Aksan I., Goding C.R. (1998). Targeting the microphthalmia basic helix–loop–helix–leucine zipper transcription factor to a subset of E-box elements *in vitro* and *in vivo*. Mol. Cell. Biol..

[24] Levy C., Khaled M., Fisher D.E. (2006). MITF: Master regulator of melanocyte development and melanoma oncogene. Trends Mol. Med..

[25] Shin H., Hong S.D., Roh E., Jung S.H., Cho W.J., Park S.H., Yoon D.Y., Ko S.M., Hwang B.Y., Hong J.T. (2015). cAMP-dependent activation of protein kinase A as a therapeutic target of skin hyperpigmentation by diphenylmethylene hydrazinecarbothioamide. Br. J. Pharmacol..

[26] Lee J., Jung E., Huh S., Boo Y.C., Hyun C.G., Kim Y.S., Park D. (2007). Mechanisms of melanogenesis inhibition by 2,5-dimethyl-4-hydroxy-3(2*H*)-furanone. Br. J. Dermatol..

[27] An S.M., Lee S.I., Choi S.W., Moon S.W., Boo Y.C. (2008). *p*-Coumaric acid, a constituent of *Sasa quelpaertensis* Nakai, inhibits cellular melanogenesis stimulated by α-melanocyte stimulating hormone. Br. J. Dermatol..

[28] Saha B., Singh S.K., Sarkar C., Bera R., Ratha J., Tobin D.J., Bhadra R. (2006). Activation of the MITF promoter by lipid-stimulated activation of p38-stress signalling to CREB. Pigment Cell Res..

[29] Khaled M., Larribere L., Bille K., Aberdam E., Ortonne J.P., Ballotti R., Bertolotto C. (2002). Glycogen synthase kinase 3β is activated by cAMP and plays an active role in the regulation of melanogenesis. J. Biol. Chem..

[30] Su T.R., Lin J.J., Tsai C.C., Huang T.K., Yang Z.Y., Wu M.O., Zheng Y.Q., Su C.C., Wu Y.J. (2013). Inhibition of melanogenesis by gallic acid: Possible involvement of the PI3K/AKT, MEK/ERK and Wnt/β-catenin signaling pathways in B16F10 cells. Int. J. Mol Sci..

